# Impact of preexisting diabetes mellitus on cardiovascular and all-cause mortality in patients with atrial fibrillation: A meta-analysis

**DOI:** 10.3389/fendo.2022.921159

**Published:** 2022-08-01

**Authors:** Juan Xu, Yimeng Sun, Dandan Gong, Yu Fan

**Affiliations:** ^1^ Department of Oncology, Ganyu District People’s Hospital of Lianyungang City, Lianyungang, China; ^2^ Institute of Molecular Biology and Translational Medicine, The Affiliated People’s Hospital, Jiangsu University, Zhenjiang, China

**Keywords:** diabetes mellitus, atrial fibrillation, all-cause mortality, cardiovascular mortality, meta-analysis

## Abstract

**Objective:**

To determine the impact of preexisting diabetes mellitus on cardiovascular and all-cause mortality in patients with atrial fibrillation (AF) by conducting a meta-analysis.

**Methods:**

PubMed and Embase databases were comprehensively searched for relevant studies publishing until May 19, 2022. Cohort studies or post-hoc analyses of clinical trials that investigated the association of diabetes mellitus with cardiovascular or all-cause mortality in AF patients were included.

**Results:**

A total of 21 studies with 526,136 AF patients were identified. The pooled prevalence of diabetes mellitus in patients with AF was 26%. The summary multivariable-adjusted risk ratio (RR) of all-cause mortality was 1.37 (95% confidence intervals [CIs] 1.23–1.53) for patients with diabetes versus those without diabetes. Moreover, diabetes mellitus was also associated with an increased risk of cardiovascular mortality (RR 1.46; 95% CI 1.34–1.58). Stratified analyses suggested that the impact of diabetes on all-cause and cardiovascular mortality was consistently observed in each named subgroup.

**Conclusion:**

The presence of diabetes mellitus in patients with AF is associated with an increased risk of cardiovascular and all-cause mortality, even after adjustment for important confounding factors.

## Introduction

Atrial fibrillation (AF) is the most common type of heart rhythm disorder that can result in stroke, heart failure, myocardial infarction, and venous thromboembolism (1). The healthcare burden of atrial fibrillation is increasing because of the accelerated aging of the population worldwide (2). Despite direct-acting oral anticoagulants effectively reducing stroke risk (3), atrial fibrillation was still associated with a 1.95-fold increased risk of all-cause mortality (4). Therefore, risk stratification of survival outcomes in atrial fibrillation patients is an urgent demand for better clinical management.

Diabetes mellitus is a well-established risk factor for the development of atrial fibrillation. Patients with diabetes had approximately 28% higher risk of atrial fibrillation as compared with those without diabetes (5). Diabetes mellitus is associated with an increased risk of mortality (6). The presence of diabetes mellitus in atrial fibrillation patients may reinforce the risk of mortality. The effect of diabetes mellitus on adverse outcomes has been extensively investigated in atrial fibrillation patients (7). However, conflicting results have been reported on the association of diabetes with all-cause or cardiovascular mortality in patients with atrial fibrillation (8–11).

No previous meta-analysis has evaluated the association of diabetes mellitus with survival outcomes in patients with atrial fibrillation. To address these knowledge gaps, we performed this meta-analysis to determine the impact of diabetes mellitus on cardiovascular and all-cause mortality in atrial fibrillation patients.

## Methods

### Data source and literature search

We performed this meta-analysis according to the guideline of the Preferred Reporting Items for Systematic Reviews and Meta-analyses statement (12). Two authors independently searched PubMed and Embase databases until May 19, 2022, using the following combined keywords: ‘atrial fibrillation’ AND ‘diabetes’ OR ‘diabetic’ AND ‘survival’ OR ‘death’ OR ‘mortality’ AND ‘follow-up’ OR ‘follow up’ (**Supplementary Text S1**). Moreover, we also searched the reference lists of pertinent articles for any missing articles.

### Study selection

Inclusion criteria were as follows: 1) population: participants with a diagnosis of atrial fibrillation; 2) predictor: diabetes mellitus; 3) comparison: the presence of diabetes mellitus compared with those without diabetes; 4) outcomes: reported multivariable-adjusted relative risk of cardiovascular or all-cause mortality; and 5) study design: cohort studies or post-hoc analysis of clinical trials. For articles overlapping patients with another study, we only selected the article with the most complete data or the longest follow-up duration. The exclusion criteria included the following: 1) outcome measures were not of interest, 2) duplicate studies, and 3) an adjusted risk estimate was not available.

### Data extraction and quality evaluation

Data extraction and quality evaluation were performed by two independent authors, and disagreements were resolved through discussion. Data collected from each study included the following: the first author’s name, publication year, country, design of the study, sample sizes, gender, mean median age of patients, anticoagulant therapy, the prevalence of diabetes mellitus, outcome measures, length of follow-up, fully adjusted risk summary for diabetes versus without diabetes, and variables adjusted. To evaluate the methodological quality of included studies, we adopted a 9-point Newcastle–Ottawa Scale (NOS) for the cohort studies (13). A study with an overall score of 7 points or more was deemed high-quality.

### Data analysis

All statistical analyses were performed using Stata 12.0 (Stata, College Station, TX, USA). The impact of diabetes mellitus on survival outcomes was summarized by pooling a fully adjusted risk ratio (RR) and 95% confidence intervals (CIs) for diabetes mellitus versus those without diabetes. Between studies, heterogeneity was explored using the *I* ^2^ statistics and Cochrane Q test. A random-effects model was utilized when there was significant heterogeneity (*I* ^2^ statistic >50% and/or *p-*value of Cochrane Q test <0.10); otherwise, we used a fixed-effects model. Publication bias was evaluated using Begg’s test, Egger’s test, and funnel plot. In case of publication bias, we run a trim-and-fill analysis to observe the impact of publication bias. Sensitivity analyses were performed by the exclusion of individual studies each time to recalculate the pooling risk summary. Subgroup analyses were conducted according to the study design, geographical region, type of atrial fibrillation, sample size, mean/median age of patients, and follow-up duration.

## Results

### Search results and study characteristics

**Figure 1:**
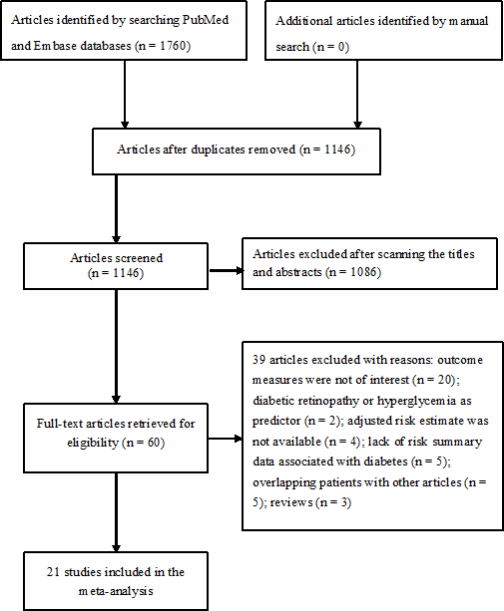
Flowchart showing the process of study selection.

Briefly, a total of 1,760 articles were identified in our initial literature search, of which 614 duplicates were removed, and 1,086 articles were further excluded after scanning the titles or abstracts. Sixty full-text articles were retrieved for detailed assessment. Finally, 21 studies (8–11, 14–30) were included in the meta-analysis after applying the predefined criteria (**Figure 1**).

The main features of these eligible studies are summarized in **Table 1**. The included studies were published between 2011 and 2022. Three studies (19, 21, 27) were post-hoc analyses of randomized controlled trials, five studies (11, 14, 17, 26, 28) were retrospective designs, and others were prospective studies. Sample sizes ranged between 278 and 326,832, with 526,136 atrial fibrillation patients. The median/mean duration of follow-up ranged from 12 months to 6.3 years. **Supplementary Table S1** describes the comorbidities and concomitant treatment of the included studies. Based on the criteria of the NOS, all the included studies were deemed to have high quality (**Supplementary Table S2**).

**Table 1:** Baseline characteristic of the included studies.

Author/year	Region	Study design	Patients (% men)	Age (years)	Anticoagulant therapy	Prevalence of diabetes	Outcomes Relative risk	Follow-up	Maximum adjusted variables
Ehrlich 2011 (8)	Germany	P	AF 278 (63)	70 ± 11	NR	26.3%	Total death1.56 (0.87–2.80)	1.3 years	Age, sex, hypertension, LVEF, CHADS2 score, previous stroke or TIA, implantable cardioverter/defibrillator, or pacemaker
Melgaard 2014 (14)	Denmark	R	Non-valvular AF 73,799 (64.3)	Mean 62.8	44.7% VKA	11.2%	Total death 2.02 (1.94–2.12) #	5 years	Female, prior stroke, HF, hypertension, vascular disease, VKA treatment
Inoue 2014 (9)	Japan	P	Non-valvular AF 7,406 (64.3)	70.2 ± 9.9	86.5% warfarin	18.3%	Total death 1.02 (0.67–1.56) CV death 1.33 (0.71–2.49)	2 years	Age, sex, CHADS, CAD, cardiomyopathy, HF, hypertension, medications
Huang 2015 (15)	China	P	Non-valvular AF 1,644 (48.3)	70 ± 12.9	12.5% warfarin	16.8%	Total death 1.56 (1.13–2.16) CV death 1.62 (1.05–2.48)	12 months	Age, sex, weight, type of AF, stroke or TIA, HF, COPD, heart rate, ARB, lipid-lowering agents
Vılchez 2015 (18)	Spain	P	AF 562 (51)	77 (71–82)	100% anticoagulant	28.0%	Total death 1.76 (1.08–2.88)	4.3 years	Age, history of stroke or TIA, CAD, renal failure, CHA2DS2-VASc score, soluble suppression of tumorigenicity-2
Pastori 2015 (16)	Italy	P	AF 837 (56.4)	73.2 ± 8.5	100% VKA	19.8%	CV death 2.43 (1.33–4.43)	2.5 years	Age, sex, smoking, hypertension, MI, stroke/TIA, HF, use of antiplatelet agents and statins, 11-dehydro-thromboxane B2
Senoo 2016 (10)	Japan	P	AF 1,791 (50.5)	81.8 ± 5.3	55.4% OAC	22.3%	Total death 1.12 (0.85–1.45)	12 months	Age, sex, hypertension, HF, stroke, vascular disease, use of oral anticoagulation
Pokorney 2016 (19)	Multination	*Post hoc*	AF 14,171 (60.4)	Median 73	100% anticoagulant	39.8%	Total death 1.45 (1.28–1.67) CV death 1.44 (1.24–1.68)	1.9 years	Age, sex, race, ethnicity, region, heart rate, BMI, SBP, DBP, years of AF diagnosis, type of AF, stroke or TIA, HF, hypertension, creatinine clearance, creatinine, PAD, COPD, gastrointestinal bleeding, liver disease, alcohol, obstructive sleep apnea, left bundle branch block
Chamberlain 2017 (11)	India	R	AF 1,430 (48.6)	73.6 ± 13.8	NR	30.6%	Total death 1.04 (0.90–1.21)	6.3 years	Age, sex hyperlipidemia, hypertension, HF, CKD, smokers, substance abuse, CAD, stroke, cancer, COPD, depression, dementia, osteoporosis, anxiety, schizophrenia
Echouffo-Tcheugui 2017 (20)	USA	P	AF 9,749 (57.4)	75 (67–82)	76.4% OAC	29.5%	Total death 1.28 (1.12–1.46) # CV death 1.31 (1.09–1.58) #	2.41 years	Age, race, use of anticoagulants, eGFR, type of AF, history of ablation, pulse pressure
Karayiannides 2018 (17)	Sweden	R	Non-valvular AF 326,832 (55.3)	74.7 ± 12.3	43.6% warfarin	17.7%	Total death 1.28 (1.25–1.31)	3.7 years	Age, sex, comorbidities, medications
Perera 2018 (21)	Multination	*Post hoc*	AF 7,554 (58)	71 ± 10	NR	19%	Total death 1.4 (1.3–1.6)	3.7 years	Age, BMI, race, stroke/TIA, heart rate, CAD, DBP, hemoglobin, eGFR, LVSD, antiplatelet therapy
Wändell 2018 (22)	Sweden	R	AF 12,283 (54.1)	≥45	NR	19.6%	Total death 1.12 (1.02–1.22) #	5.8 years	Age, educational level, marital status, neighborhood socio-economic status, co-morbidities, anticoagulant treatment
Pastori 2019 (23)	Italy	P	AF 5,215 (54.6)	75 ± 9.6	74.3% VKA, 25.7% NOAC	20.1	Total death 1.32 (1.04–1.67)	1.6 years	Age, sex, CKD, active cancer, HF, pulmonary disease, PAD, previous cardiovascular disease, medications
Polovina 2020 (24)	Serbia	P	AF 1,803 (61)	69 ± 12	92.9% OAC	22%	Total death 1.56 (1.22–2.01) CV death 1.48 (1.34–1.93)	5.0 years	Age, sex, SBP, DBP, BMI, obesity, heart rate, HbA1c, cardiovascular history, LVEF, COPD, smoker, alcohol, hyperthyroidism, hypothyroidism, CKD, AF medications, antidiabetic agents
García-Fernández 2020 (25)	Spain	P	AF 1,956 (56)	73.8 ± 9.5	75.8% VKA, 24.2% DOAC	29.3%	CV death 1.73 (1.07–2.80)	2.95 years	Age, Charlson index, heart failure, HAS-BLED score
Oba 2020 (26)	Japan	R	AF 389 (49.9)	80 (74–85)	62% OAC	31.6%	Total death 1.48 (1.02–2.13) CV death 2.05 (1.32–3.17)	3.8 years	Age, sex, hypertension, hypercholesterolemia, CAD, stroke, HF, PAD, sustained AF, use of OAC at baseline
Papazoglou 2021 (27)	Greece	*Post hoc*	AF 1,109 (54.3)	73.6 ± 10.9	25.4% VKA, 47.1% DOAC	33.6%	Total death 1.40 (1.11–1.75) CV death 1.39 (1.07–1.81)	2.6 years	Age, sex, AF subtype, BMI, prior stroke or CAD, use of anticoagulants, eGFR, ACEI-ARB, rate control medication after discharge
Kezerle 2021 (28)	Israel	R	Non-valvular AF 44,451 (47.5)	75 (65–83)	24.4% warfarin, 16.2% DOAC	39.9%	Total death 1.47 (1.41–1.58)	3.2 years	Age, sex, socioeconomic status, BMI, eGFR, hypertension, HF, previous stroke or TIA, vascular disease, any oral anticoagulant
Ding 2022 (29)	Europe	P	AF 11,028 (59.3)	71 (63–77)	86.7% anticoagulant	23%	Total death 1.28 (1.08–1.52) CV death 1.41 (1.09–1.83)	2 years	Age, sex, eGFR, COPD, CAD, HF, hypercholesterolaemia, hypertension, PAD, previous hemorrhagic event, thromboembolism, sleep apnea, use of anticoagulation.
Hammoudeh 2022 (30)	Jordan	P	Non-valvular AF 1,849 (46.9)	68.4 + 12.9	81.1% anticoagulant	44.7%	Total death 1.5 (1.1–2.2)	12 months	Age, BMI, CAD, HF, use of OAC, major bleeding, enrolled as in-patient

P, prospective; R, retrospective; NR, not reported; AF, atrial fibrillation; OAC, oral anticoagulation; NOAC, non-vitamin K antagonist oral anticoagulants; DOAC, direct oral anticoagulant; VKA, vitamin K antagonists; BMI, body mass index; SBP, systolic blood pressure; DBP, diastolic blood pressure; LVEF, left ventricular ejection fraction; CKD, chronic kidney disease; LVH, left ventricular hypertrophy; LVSD, left ventricular systolic dysfunction; HF, heart failure; MI, myocardial infarction; CAD, coronary artery disease; PAD, peripheral arterial disease; COPD, chronic obstructive pulmonary disease; HbA1c, glycated hemoglobin; NT-proBNP, N-terminal pro-B-type natriuretic peptide; eGFR, estimated glomerular filtration rate; TIA, transient ischemic attack; ACE/ARB, angiotensin-converting enzyme/angiotensin-receptor blockers; CV, cardiovascular disease.

^#^Results pooled from subgroup using a fixed-effects model.

### Prevalence of diabetes mellitus in atrial fibrillation patients

**Figure 2:**
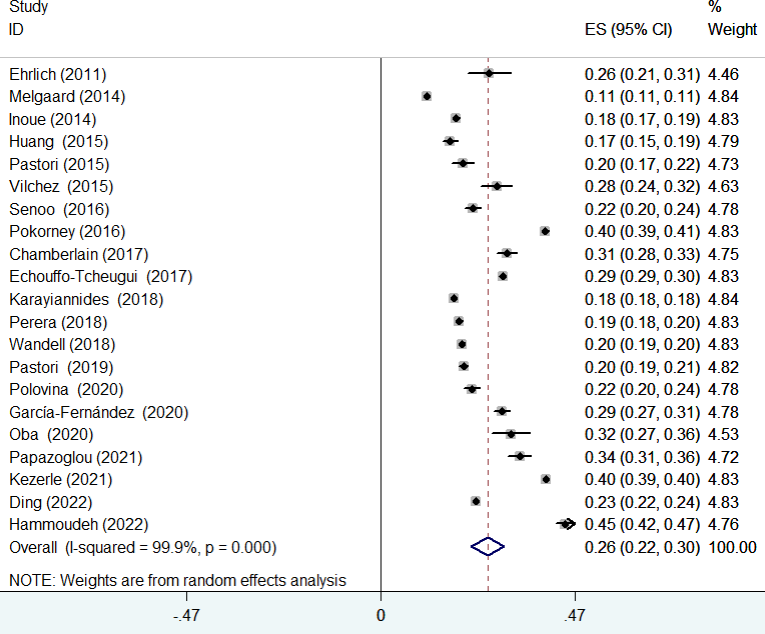
Pooled prevalence of diabetes mellitus in patients with atrial fibrillation.

The prevalence of diabetes mellitus ranged from 11.2% to 44.7%. As shown in **Figure 2**, the pooled prevalence of diabetes mellitus across the included studies was 0.26 (95% CI 0.22–0.30) in a random-effects model with significant heterogeneity (*I* ^2^ = 99.9%; *p* < 0.001).

### All-cause mortality

**Figure 3:**
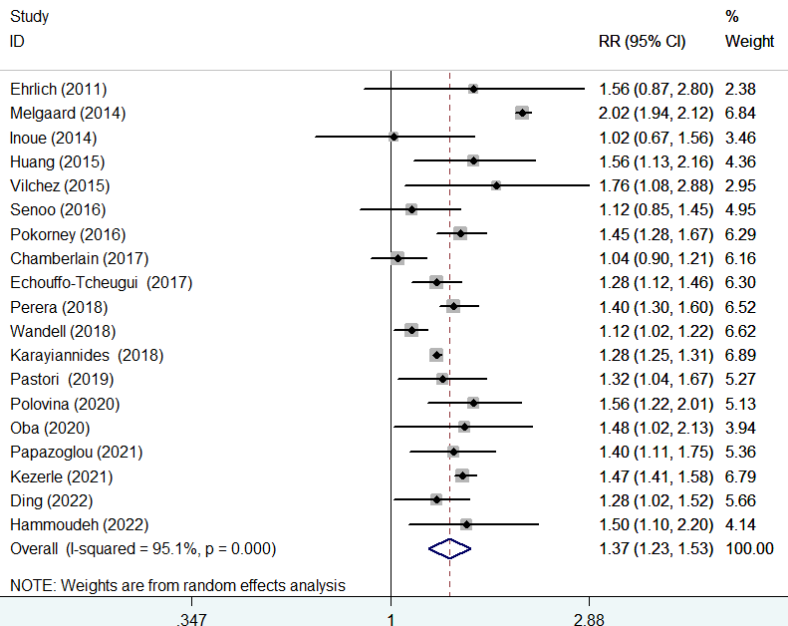
Forest plot showing the pooled risk ratio (RR) with 95% CI of all-cause mortality for those with diabetes versus those without diabetes.

The association of diabetes mellitus with all-cause mortality was reported in 19 studies (8–11, 14, 15, 17–24, 26–30). A random-effects model meta-analysis indicated that diabetes mellitus was associated with an increased risk of all-cause mortality (RR 1.37; 95% CI 1.23–1.53; *I* ^2^ = 95.1%, *p* < 0.001; **Figure 3**) compared with those without diabetes. After a very large study (17) was removed, the pooled RR of all-cause mortality was 1.38 (95% CI 1.22–1.57). Leave-one-out study sensitivity analysis confirmed the robustness of the summary risk estimate (data not shown). There was no evidence of publication bias according to the results of Begg’s test (*p* = 0.441), Egger’s test (*p* = 0.964), and symmetry of the funnel plot (**Supplementary Figure S1**). Additionally, significant associations of diabetes mellitus with all-cause mortality were consistently observed in each subgroup (**Table 2A**).

**Table 2:** Subgroup analyses on all-cause (A) and cardiovascular (B) mortality.

Subgroup	Number of studies	Pooled risk ratio	95% confidence intervals	Heterogeneity between studies
**A. All-cause mortality**				
Study design				
Prospective Retrospective Post-hoc analysis	10 6 3	1.33 1.31 1.42	1.23–1.44 1.11–1.68 1.31–1.53	*p* = 0.527; *I* ^2^ = 0.0% *p* < 0.001; *I* ^2^ = 98.6% *p* = 0.915; *I* ^2^ = 0.0%
Geographical region				
Asia Others	6 12	1.40 1.37	1.20–1.56 1.16–1.63	*p* = 0.242; *I* ^2^ = 25.6% *p* < 0.001; *I* ^2^ = 97.2%
Atrial fibrillation type				
Non-valvular type All types	5 14	1.54 1.29	1.23–1.93 1.21–1.37	*p* < 0.001; *I* ^2^ = 95.2% *p* = 0.007; *I* ^2^ = 55.2%
Sample sizes				
≥3,000 <3,000	10 9	1.39 1.36	1.18–1.58 1.18–1.57	*p* < 0.001; *I* ^2^ = 97.4% *p* = 0.037; *I* ^2^ = 51.3%
Mean/median age				
≥75 years <75 years	6 13	1.37 1.38	1.25–1.51 1.18–1.60	*p* = 0.159; *I* ^2^ = 37.0% *p* < 0.001; *I* ^2^ = 96.7%
Follow-up duration				
≥3 years <3 years	8 11	1.43 1.29	1.18–1.75 1.26–1.32	*p* < 0.001; *I* ^2^ = 96.7% *p* = 0.571; *I* ^2^ = 0.0%
**B. Cardiovascular mortality**				
Study design				
Prospective Post-hoc analysis	7 2	1.45 1.43	1.30–1.61 1.25–1.63	*p* = 0.555; *I* ^2^ = 0.0% *p* = 0.820; *I* ^2^ = 0.0%
Geographical region				
Asia Others	3 6	1.71 1.43	1.30–2.25 1.29–1.59	*p* = 0.513 *I* ^2^ = 0.0% *p* = 0.462; *I* ^2^ = 0.0%
Atrial fibrillation type				
Non-valvular type All types	2 8	1.52 1.45	1.07–2.17 1.34–1.58	*p* = 0.611; *I* ^2^ = 0.0% *p* = 0.419; *I* ^2^ = 1.3%
Sample sizes				
≥3,000 <3,000	3 7	1.39 1.55	1.25–1.55 1.37–1.76	*p* = 0.737; *I* ^2^ = 0.0% *p* = 0.533; *I* ^2^ = 0.0%
Mean/median age				
≥75 years <75 years	2 8	1.40 1.47	1.18–1.66 1.34–1.62	*p* = 0.065; *I* ^2^ = 70.6% *p* = 0.807; *I* ^2^ = 0.0%
Follow-up duration				
≥2 years <2 years	8 2	1.46 1.46	1.32–1.61 1.26–1.68	*p* = 0.413; *I* ^2^ = 2.2% *p* = 0.613; *I* ^2^ = 0.0%

### Cardiovascular mortality

**Figure 4:**
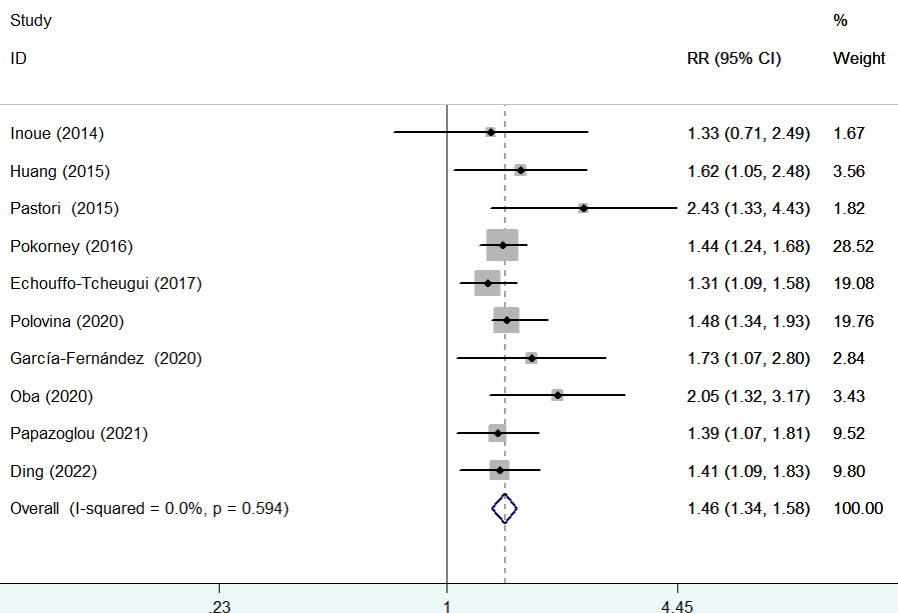
Forest plot showing the pooled risk ratio (RR) with 95% CI of major bleeding for those with diabetes versus those without diabetes.

Ten studies (9, 15, 16, 19, 20, 24–27, 29) provided data on the association of diabetes mellitus with cardiovascular mortality. A fixed-effects model meta-analysis suggested that diabetes mellitus was associated with an increased risk of cardiovascular mortality (RR 1.46; 95% CI 1.34–1.58; *I* ^2^ = 0%, *p* = 0.594; **Figure 4**) compared with those without diabetes. Leave-one-out study sensitivity analysis further confirmed the robustness of the summary risk estimate (data not shown). Furthermore, significant associations between diabetes mellitus with cardiovascular mortality were consistently observed in each subgroup (**Table 2B**). Egger’s test (*p* = 0.048) but not Begg’s test (*p* = 0.210) suggested the likelihood of publication bias. The ‘trim-and-fill’ analysis indicated that the pooling RR of cardiovascular mortality was 1.42 (95% CI 1.05–1.91; *p* < 0.001) and imputed three potentially missing studies (**Supplementary Figure S2**).

## Discussion

This is the first meta-analysis to evaluate the impact of preexisting diabetes mellitus on cardiovascular and all-cause mortality in atrial fibrillation patients. Our meta-analysis confirmed that diabetes mellitus was associated with a higher risk of cardiovascular and all-cause mortality in patients with atrial fibrillation. Atrial fibrillation patients with preexisting diabetes had a 46% higher risk of cardiovascular mortality and 37% higher risk of all-cause mortality compared with their non-diabetic counterparts. These results indicated that the presence of diabetes mellitus in atrial fibrillation patients reinforced the mortality risk.

Apart from the cardiovascular and all-cause mortality outcomes, diabetes in atrial fibrillation patients also conferred a higher risk of heart failure (17, 24), myocardial infarction (17), stroke (17, 27), and bleeding events (17). These results suggested that diabetes is an important predictor of the risk classification of AF patients.

Atrial fibrillation and heart failure frequently coexist (31). The presence of atrial fibrillation was associated with a higher risk of mortality in heart failure patients (32). Conversely, incident heart failure conferred a particularly increased risk of mortality in patients with heart failure (33). Sodium-glucose co-transporter 2 inhibitors (SGLT2i) are a class of medication approved for the management of type 2 diabetes. Treatment with SGLT2i could significantly reduce cardiovascular or all-cause mortality in heart failure in patients with or without diabetes (34, 35). Therefore, the association of diabetes with mortality outcomes in patients with atrial fibrillation may also be biased by the use of SGLT2i.

Our subgroup analysis showed that the impact of diabetes on all-cause or cardiovascular mortality was similar in patients with age ≥75 years and those with age <75 years. This finding may be correlated with inadequate anticoagulation in many elderly atrial fibrillation patients (15). Moreover, the value of diabetes in predicting all-cause or cardiovascular mortality appeared to be stronger in patients with a non-valvular type of atrial fibrillation. This result may be explained by the higher risk of stroke and the bleeding risk associated with anticoagulant treatment in the non-valvular type of atrial fibrillation (36). However, it should be noted that the results of subgroup analysis were established on a limited number of studies.

The precise mechanisms underlying the impact of diabetes mellitus on survival in atrial fibrillation patients are not fully elucidated. First, atrial fibrillation patients with diabetes had more concomitant risk factors and comorbidities (17, 25). Second, atrial fibrillation patients with diabetes had a significant reduction in the quality of anticoagulation control (25). Third, diabetes could also cause structural, electrical, electromechanical, and autonomic remodeling (37), which could be responsible for atrial fibrillation recurrence. Finally, diabetes may promote the development of cardiomyopathy and heart failure through systemic inflammation, microvascular dysfunction, and oxidative stress (38, 39).

Our meta-analysis had important clinical implications. The prevalence of diabetes mellitus was up to 39.9% among patients with atrial fibrillation in our analyzed studies. The association between diabetes mellitus and atrial fibrillation has remained under-recognized by clinicians (40). Considering atrial fibrillation patients with diabetes had reduced survival, close monitoring of blood glucose levels and intensive glycemic control are warranted for atrial fibrillation patients with diabetes. The degree of glycemic control may affect the prognosis of atrial fibrillation patients (41). However, whether management of hyperglycemia in those with diabetes improves survival outcomes requires further study.

Several limitations should be mentioned in the current meta-analysis. First, half of the included studies were retrospective in nature, and potential selection bias may have occurred. Second, significant heterogeneity was found in pooling the prevalence of diabetes and all-cause mortality. Different types of atrial fibrillation, methods of diabetes diagnosis, or duration of follow-up may partially explain the observed heterogeneity. Third, the impact of diabetes on survival outcomes may be biased by the use of different anticoagulant agents in patients with atrial fibrillation (42). Lack of adjusted information on the use of medications (including antidiabetic drugs, anticoagulation, and heart failure-directed therapy), severity or duration of diabetes, and glycemic control may confound the pooling risk summary. Finally, the analyzed studies did not distinguish the pattern of atrial fibrillation (permanent or persistent) and type of diabetes mellitus (type 1 or type 2) in patients with atrial fibrillation. Particularly, the anticoagulant therapy was not clearly reported in the included studies. Therefore, we failed to perform a subgroup analysis based on these factors.

## Conclusions

The presence of diabetes mellitus in patients with atrial fibrillation is associated with an increased risk of cardiovascular and all-cause mortality, even after adjustment for important confounding factors. Detection of blood glucose levels may improve risk stratification of survival outcomes in atrial fibrillation patients.

## Data availability statement

All data generated or analyzed during this study are included in this published article.

## Author contributions

Study conception/design and interpretation of data: DG and YF. Literature search, data extraction, quality assessment, and statistical analysis: JX and YS. Writing the manuscript: JX. Revising the manuscript: YF. All authors read and approved the final manuscript.

## Funding

This work is supported by 1) Jiangsu Provincial Key Research and Development Special Fund (CXTDC2016006, QNRC2016446), 2) Zhenjiang Key Research and Development Fund (SH2021038), and 3) Suqian Science and Technology Support Project Fund (K201907).

## Conflict of interest

The authors declare that the research was conducted in the absence of any commercial or financial relationships that could be construed as a potential conflict of interest.

## Publisher’s note

All claims expressed in this article are solely those of the authors and do not necessarily represent those of their affiliated organizations, or those of the publisher, the editors and the reviewers. Any product that may be evaluated in this article, or claim that may be made by its manufacturer, is not guaranteed or endorsed by the publisher.
