# Supplementary material for: Impact of preexisting diabetes mellitus on cardiovascular and all-cause mortality in patients with atrial fibrillation: A meta-analysis
Source: Front Endocrinol (Lausanne). 2022 Aug 1;13:921159. doi: 10.3389/fendo.2022.921159 (PMC9376236; doi:10.3389/fendo.2022.921159)
Supplement: Supplementary file 1 [file DataSheet_1.docx]

**Supplemental Text S1 – Search strategy**

**Medical Databases---PubMed and Embase**

| **1. PubMed Search Strategy** | Query | Items found |
| --- | --- | --- |
| #1 | Search “atrial fibrillation” | 98100 |
| #2 | Search “**diabetes**” | 872,565 |
| #3 | Search “mortality**”** | 1,442,901 |
| #4 | Search “death” | 1,111,015 |
| #5 | Search “survival” | 2,407,074 |
| #6 | Search #3 OR #4 OR #5 | 2,856,009 |
| #7 | Search “follow-up” | 1,490,552 |
| #8 | Search “follow up” | 1,369,575 |
| #9 | Search #7 OR #8 | 1,730,438 |
| #10 | Search #1 AND #2 AND #6 AND #9 | 1203 |
| #11 | Filters: **Humans** | 1089 |
|  |  |  |
|  |  |  |
|  |  |  |
| **2. Embase Search Strategy** | Query | Items found |
| #1 | 'atrial fibrillation':ti | 77,432 |
| #2 | 'diabetes'/exp OR diabetes | 1,422,734 |
| #3 | 'diabetic'/exp OR diabetic | 1,206,220 |
| #4 | #2 or #3 | 1,461,278 |
| #5 | 'survival'/exp OR survival | 2,033,171 |
| #6 | 'mortality'/exp OR mortality | 1,767,793 |
| #7 | 'death'/exp OR death | 1,674,686 |
| #8 | #5 or #6 or #7 | 4,444,955 |
| #9 | 'follow up'/exp OR 'follow up' OR (follow AND up) | 2,336,249 |
| #10 | 'follow-up'/exp OR 'follow-up' | 2,324,199 |
| #11 | #9 OR #10 | 2,336,249 |
| #12 | #1 AND #4 AND #8 AND #11 | 1,309 |
| #13 | #12 AND ('article'/it OR 'article in press'/it OR 'letter'/it) AND [humans]/lim | 671 |

Additionally, a manual search was performed in the reference lists of pertinent articles.
