# Supplementary material for: Impact of preexisting diabetes mellitus on cardiovascular and all-cause mortality in patients with atrial fibrillation: A meta-analysis
Source: Front Endocrinol (Lausanne). 2022 Aug 1;13:921159. doi: 10.3389/fendo.2022.921159 (PMC9376236; doi:10.3389/fendo.2022.921159)

**Supplemental Figure S1** Funnel plot showing the association of diabetes with all-cause mortality. Each study is represented by one circle.


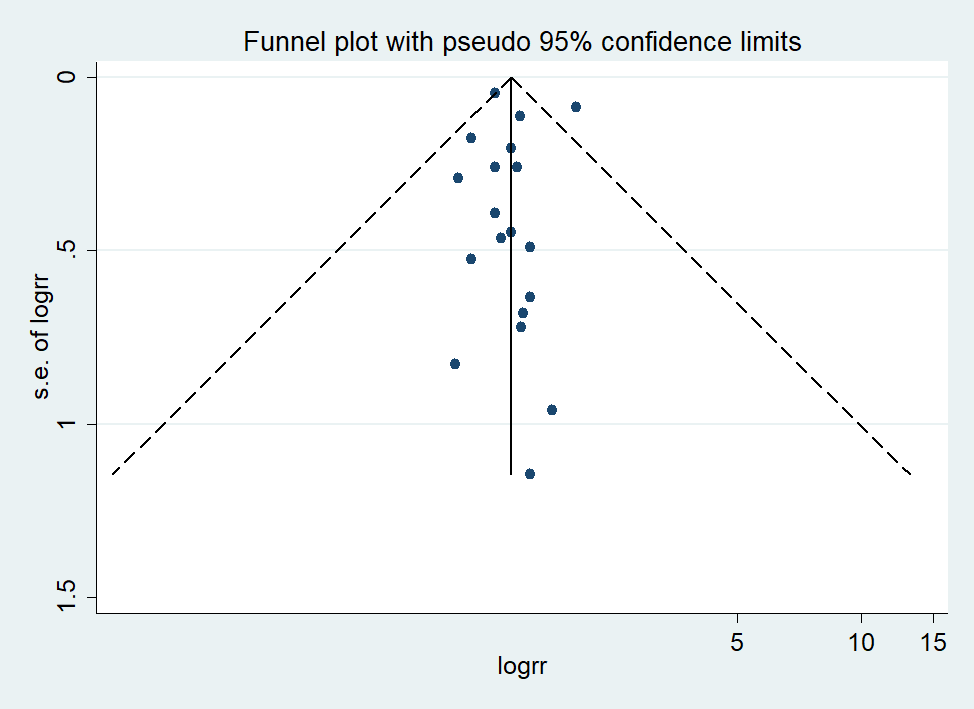


**Supplemental Figure S2** Funnel plot showing the association of diabetes with cardiovascular mortality. The circles alone are real studies and the circles enclosed in boxes are ‘filled’ studies.


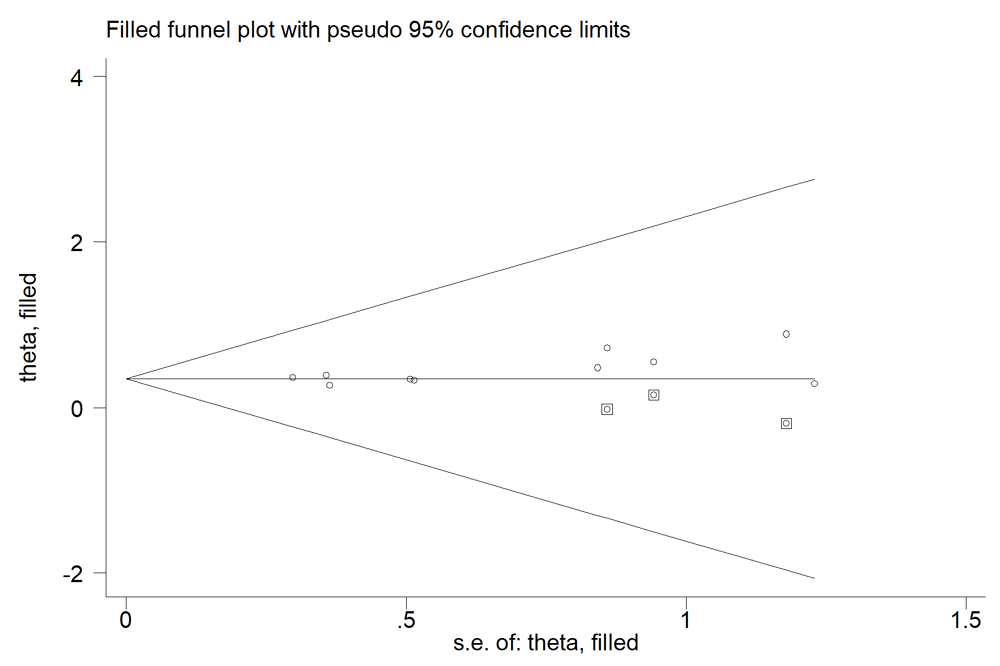

Supplement: Supplementary file 2 [file DataSheet_2.docx]
