# Supplementary material for: Impact of preexisting diabetes mellitus on cardiovascular and all-cause mortality in patients with atrial fibrillation: A meta-analysis
Source: Front Endocrinol (Lausanne). 2022 Aug 1;13:921159. doi: 10.3389/fendo.2022.921159 (PMC9376236; doi:10.3389/fendo.2022.921159)
Supplement: Supplementary file 3 [file Table_1.doc]

Supplemental Table S1 Comorbidities and concomitant treatment of the included studies

| Author/Year | HYP (%) | Stroke or TIA (%) | HF (%) | CAD (%) | COPD (%) | RD (%) | Antiplatelet (%) | ACEIs (%) | ARBs (%) | CCBs (%) | β-blockers (%) | Statins (%) | Digoxin (%) | Diuretics (%) | ARD (%) |
| --- | --- | --- | --- | --- | --- | --- | --- | --- | --- | --- | --- | --- | --- | --- | --- |
| Ehrlich 2011 (8) | 77 | 14 | NR | 40.7 | NR | NR | NR | NR | NR | NR | NR | NR | NR | NR | NR |
| Melgaard 2014 (14) | 22.6 | 15.2 | 13.1 | NR | 6.3 | 3.8 | NR | NR | NR | NR | NR | NR | NR | NR | NR |
| Inoue 2014 (9) | 60.4 | 13.8 | 27.7 | 10.5 | NR | NR | 26.2 | NR | NR | NR | NR | NR | NR | NR | NR |
| Huang 2015 (15) | 61.4 | 19.8 | 31 | 47.2 | 12.7 | NR | 64.5 | 22.2 | 17.4 | 25.7 | 44.6 | 26.5 | 23.1 | 32.3 | NR |
| Vılchez 2015 (18) | 83 | 12 | 24 | 19 | NR | 10 | 19 | 29 | 35 | 31 | 44 | 32 | 24 | 55 | NR |
| Pastori 2015 (16) | 92.8 | NR | 17.7 | NR | NR | NR | 7 | NR | NR | NR | NR | 37.4 | NR | NR | NR |
| Senoo 2016 (10) | 63.7 | 23.7 | 33.5 | NR | NR | NR | 33.3 | NR | NR | NR | NR | NR | NR | NR | NR |
| Pokorney 2016 (19) | 90.5 | 52.4 | 62.5 | NR | 10.4 | NR | 36.6 | NR | NR | NR | NR | NR | NR | NR | NR |
| Chamberlain 2017 (11) | 71.1 | 14.6 | 18.2 | 39 | 34.4 | 20.2 | NR | NR | NR | NR | NR | NR | NR | NR | NR |
| Echouffo-Tcheugui 2017 (20) | 83.1 | 16 | 32.9 | 36.3 | 16.5 | 37.2 | 44.3 | 35.5 | 17.8 | 30.4 | 64.3 | 55.4 | 23.6 | NR | NR |
| Karayiannides 2018 (17) | 48.5 | 17 | 29.5 | 30.1 | NR | 4.6 | 54.9 | NR | NR | NR | 70.7 | 30.9 | 20.8 | 49.2 | NR |
| Perera 2018 (21) | 85 | 13 | 33 | 26 | NR | NR | 17 | NR | NR | NR | NR | 25.5 | NR | NR | NR |
| Wändell 2018 (22) | 45.5 | 20.9 | 46.3 | 26.3 | NR | NR | NR | NR | NR | NR | NR | NR | NR | NR | NR |
| Pastori 2019 (23) | 80.2 | 16.4 | 15.5 | NR | NR | 45.2 | 12.4 | 28.8 | 22.8 | 23 | 52.6 | 34.6 | 9.2 | 36 | 25.1 |
| Polovina 2020 (24) | 82.1 | 9.4 | 28.6 | 28.2 | NR | 21.2 | 16.6 | NR | NR | 27.1 | 56.1 | 43 | 7.2 | 30.7 | NR |
| García-Fernández 2020 (25) | 80.4 | 16.9 | 28.3 | 18.2 | NR | 19.3 | NR | 30.8 | 40.6 | 75.9 | 60.3 | 55 | 18.1 | 57.4 | 24.6 |
| Oba 2020 (26) | 78.1 | 45 | 63.8 | 22.9 | NR | NR | NR | NR | NR | NR | NR | NR | NR | NR | NR |
| Papazoglou 2021 (27) | 80.1 | 14.8 | 49.9 | NR | 12.8 | 14.6 | 19.7 | NR | NR | NR | NR | NR | NR | NR | 36.9 |
| Kezerle 2021 (28) | 77.3 | 12.5 | 18.6 | NR | NR | NR | NR | NR | NR | NR | NR | NR | NR | NR | NR |
| Ding 2022 (29) | 61.6 | 6.1 | 39.2 | 27.6 | 8.8 | NR | 20 | 42.7 | 18.9 | 16.8 | 62.1 | 42 | 14.5 | 51.4 | 27.5 |
| Hammoudeh 2022 (30) | 76.2 | 11.2 | 23.9 | 11.1 | NR | 9 | 46 | 40.1 | NR | 11.4 | 79.7 | 38.7 | NR | 38.5 | NR |

**Abbreviations:** NR, not reported; HPY, hypertension; HF, heart failure; COPD, chronic obstructive pulmonary disease; RD, renal disease; ACEIs, angiotensin-converting enzyme inhibitors; ARBs, angiotensin-renin blockers; CCBs, calcium channel blockers; ARD, antiarrhythmic drugs.
