# Supplementary material for: Impact of preexisting diabetes mellitus on cardiovascular and all-cause mortality in patients with atrial fibrillation: A meta-analysis
Source: Front Endocrinol (Lausanne). 2022 Aug 1;13:921159. doi: 10.3389/fendo.2022.921159 (PMC9376236; doi:10.3389/fendo.2022.921159)
Supplement: Supplementary file 4 [file Table_2.doc]

~~Supplemental Table S2 Methodological quality of the included studies~~

| Author/year | Representativeness of the exposed cohort | Selection of the non-exposed cohort | Ascertainment of exposure | Demonstration that outcome was not present at study start | Comparability of cohorts based on the design or analysis | Assessment of outcome | Enough follow-up periods (≥3 years) | Adequacy of follow-up of cohorts | Overall Newcastle-Ottawa Scale scores |
| --- | --- | --- | --- | --- | --- | --- | --- | --- | --- |
| Ehrlich 2011 (8) | ★ | ★ | ★ | ★ | ★ | ★ |  | ★ | 7 |
| Melgaard 2014 (14) |  | ★ | ★ | ★ | ★★ | ★ | ★ | ★ | 8 |
| Inoue 2014 (9) |  | ★ | ★ | ★ | ★★ | ★ |  | ★ | 7 |
| Huang 2015 (15) |  | ★ | ★ | ★ | ★★ | ★ |  | ★ | 7 |
| Vılchez 2015 (18) | ★ | ★ | ★ | ★ | ★ | ★ | ★ | ★ | 8 |
| Pastori 2015 (16) | **★** | **★** | **★** | **★** | **★**★ | **★** |  | **★** | 8 |
| Senoo 2016 (10) | ★ | ★ | ★ | ★ | ★★ | ★ |  | ★ | 8 |
| Pokorney 2016 (19) | ★ | ★ | ★ | ★ | ★★ | ★ |  | ★ | 8 |
| Chamberlain 2017 (11) | ★ | ★ | ★ | ★ | ★ | ★ | ★ | ★ | 8 |
| Echouffo-Tcheugui 2017 (20) | ★ | ★ | ★ | ★ | ★★ | ★ |  | ★ | 8 |
| Karayiannides 2018 (17) |  | ★ | ★ | ★ | ★★ | ★ | ★ | ★ | 8 |
| Perera 2018 (21) | ★ | ★ | ★ | ★ | ★ | ★ | ★ | ★ | 8 |
| Wändell 2018 (22) | ★ | ★ | ★ | ★ | ★ | ★ | ★ | ★ | 8 |
| Pastori 2019 (23) | ★ | ★ | ★ | ★ | ★★ | ★ | ★ |  | 8 |
| Polovina 2020 (24) | ★ | ★ | ★ | ★ | ★★ | ★ | ★ | ★ | 9 |
| García-Fernández 2020 (25) | ★ | ★ | ★ | ★ | ★★ | ★ |  | ★ | 8 |
| Oba 2020 (26) | ★ | ★ | ★ | ★ | ★ | ★ | ★ |  | 7 |
| Papazoglou 2021 (27) | ★ | ★ | ★ | ★ | ★★ | ★ |  | ★ | 8 |
| Kezerle 2021 (28) |  | ★ | ★ | ★ | ★★ | ★ | ★ | ★ | 8 |
| Ding 2022 (29) | ★ | ★ | ★ | ★ | ★★ | ★ |  | ★ | 8 |
| Hammoudeh 2022 (30) |  | ★ | ★ | ★ | ★★ | ★ |  | ★ | 7 |
